# Indirect Health Policy by Sports Media: The Positive Effects of the Live Broadcast of the FIFA World Cup in the General Population

**Published:** 2019-03

**Authors:** Behzad HEYDARPOUR, Ali SOROUSH, Farideh MORADI, Saeid KOMASI

**Affiliations:** 1. Cardiac Rehabilitation Center, Imam Ali Hospital, Kermanshah University of Medical Sciences, Kermanshah, Iran; 2. Lifestyle Modification Research Center, Imam Reza Hospital, Kermanshah University of Medical Sciences, Kermanshah, Iran; 3. Clinical Research Development Center, Imam Reza Hospital, Kermanshah University of Medical Sciences, Kermanshah, Iran

## Dear Editor-in-Chief

Today, the expansion of urbanization and the industrialization of societies and the rapid growth of technology and social transformation have encountered the general population of various communities with challenges related to adjustment and adaptability (1). Compatibility with tension and management of chronic stress caused by international political challenges, fluctuations and economic inflation, cultural problems, and family crises and social relationships in different societies are among the barriers greatest to mental health, especially in developing countries. In developed countries, the population enjoys life skills training and stress management to enhance psychological adjustment (2). On the other hand, a wide range of people uses outpatient mental health services. Mutually, in developing societies, not only education of life skills is not provided regularly (2), but people, for some reason, are less likely to directly use psychological services. The reasons are: (i) poor health literacy, (ii) lack of insight into adaptive problems caused by inefficient life skills, (iii) if availability, lack of willingness to attend training classes in order to the improved skills, (iv) fear of stigma as a crazy person, (v) lack of access to good mental health services, and (vi) the inability to pay (3).

In countries like Iran, the general population does not directly attempt to manage their chronic stresses and tensions. Therefore, mental health policymakers need to use indirect approaches welcomed by the general population. One of the suggested options is to watch popular sports events in every community. Sports festivals, including the FIFA World Cup, approximately 3.5 billion people around the world are attracted to television and radio (4). In Iran, nearly half of the 80 million people are interested in football matches, especially the FIFA World Cup. This one-month festival may indirectly have a positive impact on the sense of belonging, stress relief, cathartic release of tension, positive mood changes, and good parent-child relationship among viewers and listeners (5). Although the national matches of each country for the viewers are likely to be accompanied with some stress and aggression, watching extensive sporting events, such as the FIFA World Cup, generally has a positive effect on the community mental health. Especially in Iran, where people have poor insight into the diagnosis and treatment of psychological problems (6), the live broadcast of these events can be useful.

Therefore, health policymakers by media can eliminate defensive mode of direct therapeutic approaches through this kind of public-based interventions. In general, the expansion of health policy by the sports media may be associated with the improved mental health of the general population.

## References

[1] Srivastava K (2009). Urbanization and mental health. Ind Psychiatry J, 18 (2): 75–6. 21180479 10.4103/0972-6748.64028 PMC2996208

[2] Nasheeda A Abdullah HB Krauss SE Ahmed NB (2018). A narrative systematic review of life skills education: effectiveness, research gaps, and priorities. Int J Adolesc Youth, DOI: 10.1080/02673843.2018.1479278.

[3] Kantor V Knefel M Lueger-Schuster B (2017). Perceived barriers and facilitators of mental health service utilization in adult trauma survivors: A systematic review. Clin Psychol Rev, 52: 52–68. 28013081 10.1016/j.cpr.2016.12.001

[4] Fédération Internationale de Football Association (2015). 2014 FIFA World Cup™ reached 3.2 billion viewers, one billion watched final. https://www.fifa.com/worldcup/news/2014-fifa-world-cuptm-reached-3-2-billion-viewers-one-billion-watched--2745519

[5] Pringle A (2004). Can watching football be a component of developing a state of mental health for men? J R Soc Promot Health, 124 (3): 122–8. 15195452 10.1177/146642400412400313

[6] Soltani S Takian A Akbari Sari A (2017). Cultural barriers in access to healthcare services for people with disability in Iran: A qualitative study. Med J Islam Repub Iran, 31: 51. 29445680 10.14196/mjiri.31.51 PMC5804431

